# The application of peroxidase mimetic nanozymes in cancer diagnosis and therapy

**DOI:** 10.3389/fphar.2024.1339580

**Published:** 2024-01-25

**Authors:** Amin Moradi Hasan-Abad, Atefe Shabankare, Amir Atapour, Gholam Ali Hamidi, Mahmoud Salami Zavareh, Ali Sobhani-Nasab

**Affiliations:** ^1^ Autoimmune Diseases Research Center, Shahid Beheshti Hospital, Kashan University of Medical Sciences, Kashan, Iran; ^2^ Islamic Azad University, Tehran Medical Sciences Branch, Tehran, Iran; ^3^ Department of Medical Biotechnology, School of Advanced Medical Sciences and Technologies, Shiraz University of Medical Sciences, Shiraz, Iran; ^4^ Physiology Research Center, Institute for Basic Sciences, Kashan University of Medical Sciences, Kashan, Iran

**Keywords:** nanozyme, tumor, diagnosis, cancer therapy, peroxidase

## Abstract

In recent decades, scholarly investigations have predominantly centered on nanomaterials possessing enzyme-like characteristics, commonly referred to as nanozymes. These nanozymes have emerged as viable substitutes for natural enzymes, offering simplicity, stability, and superior performance across various applications. Inorganic nanoparticles have been extensively employed in the emulation of enzymatic activity found in natural systems. Nanoparticles have shown a strong ability to mimic a number of enzyme-like functions. These systems have made a lot of progress thanks to the huge growth in nanotechnology research and the unique properties of nanomaterials. Our presentation will center on the kinetics, processes, and applications of peroxidase-like nanozymes. In this discourse, we will explore the various characteristics that exert an influence on the catalytic activity of nanozymes, with a particular emphasis on the prevailing problems and prospective consequences. This paper presents a thorough examination of the latest advancements achieved in the domain of peroxidase mimetic nanozymes in the context of cancer diagnosis and treatment. The primary focus is on their use in catalytic cancer therapy, alongside chemotherapy, phototherapy, sonodynamic therapy, radiation, and immunotherapy. The primary objective of this work is to offer theoretical and technical assistance for the prospective advancement of anticancer medications based on nanozymes. Moreover, it is anticipated that this will foster the investigation of novel therapeutic strategies aimed at achieving efficacious tumor therapy.

## 1 Introduction

**FIGURE 1:**
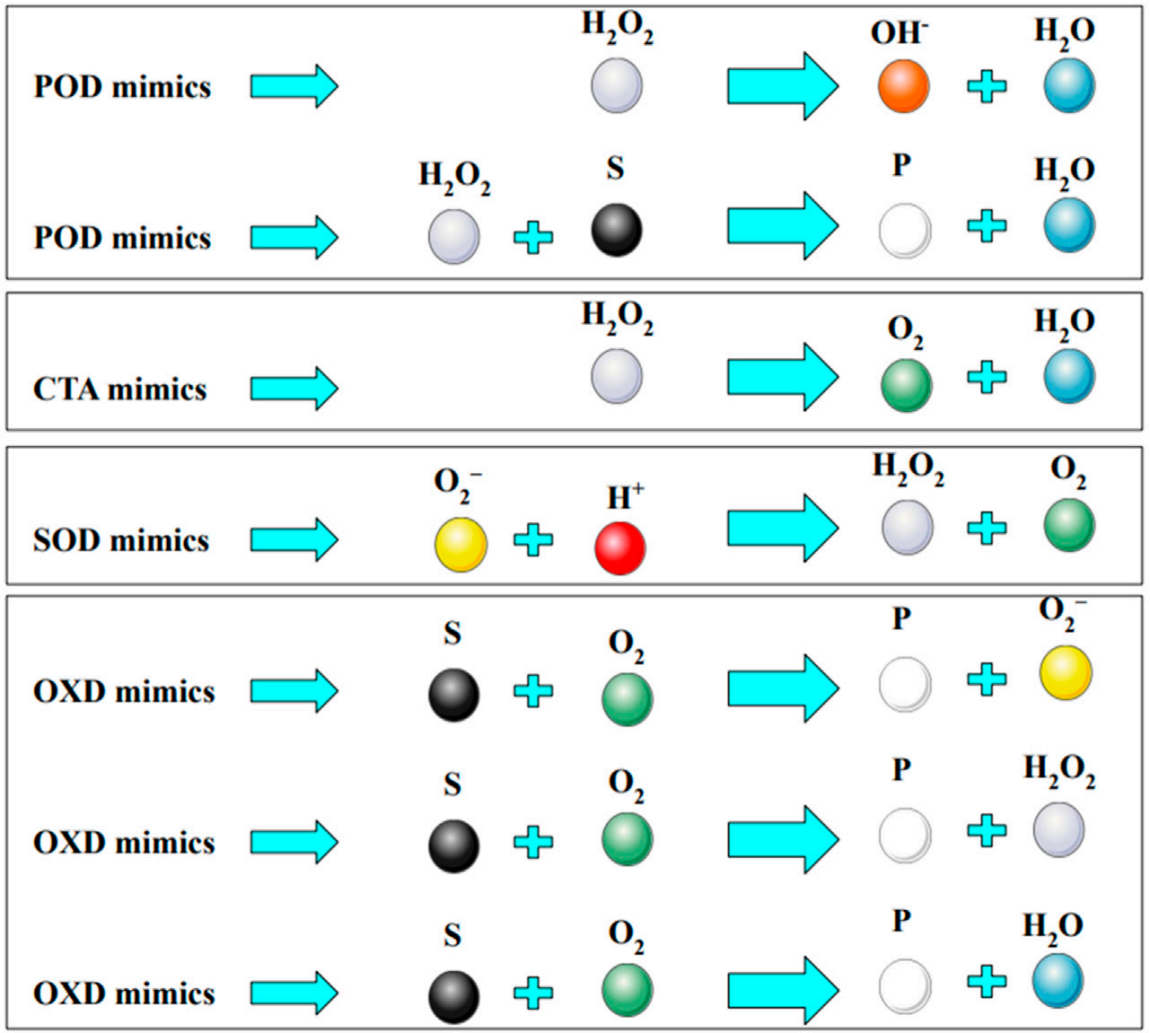
Schematic representing the catalytic reaction mediated by different nanozymes. Various nanozymes include catalase (CAT), superoxide dismutase (SOD), oxidase (OXD), and peroxidase (POD). The chemical species that enzymes interact with are known as substrates (S), and the molecules that enzyme-catalyzed reactions produce are known as products (P).

Cancer is well recognized as a prominent contributor to human mortality (Sung et al., 2021; Malehmir et al., 2023a; Jafari et al., 2023). The uncontrolled proliferation of malignant cells and their spread to other parts of the human body are linked to a variety of disorders known as cancer (Keyvani et al., 2022; Mohammadi M. et al., 2023; Malehmir et al., 2023b). One of the most significant obstacles in cancer therapy is the recurrence of tumors, the spread of malignancies or metastases, and the development of resistance to chemotherapy treatments (Bukowski et al., 2020; Riggio et al., 2021; Hasan-Abad et al., 2022; Mohammadi AH. et al., 2023). Radiotherapy, chemotherapy, and surgery are presently regarded as the most effective therapeutic methods. However, these approaches are not without notable limitations, such as the potential harm inflicted on healthy cells, the possibility of tumor recurrence, suboptimal visibility, and the incidence of tumor hypoxia (Malehmir et al., 2023a; Amjad et al., 2020; Majeed and Gupta, 2020; Dai Phung et al., 2020). There is a pressing need for the development of effective therapeutic and diagnostic procedures to improve the effectiveness of cancer treatment. In recent times, there has been a notable emphasis on the exploration of nanomaterial-mediated phototherapies, specifically photothermal therapy (PTT) and photodynamic therapy (PDT), as prospective treatment techniques for a variety of diseases, including cancer (Xiao et al., 2020) and bacterial infections (Xiao et al., 2021), and others (Xiao et al., 2018; Korupalli et al., 2020; Thangudu et al., 2020; Thangudu et al., 2021). PTT is predicated upon the utilization of photothermal heat, while PDT primarily relies on the generation and subsequent action of reactive oxygen species (ROS). When compared to conventional therapeutic methods, PDT exhibits a high degree of selectivity, can be remotely regulated, demonstrates minimal levels of systemic toxicity, and is considered a noninvasive approach (Feng et al., 2020; Hamblin, 2020; Moradi Hasan-Abad et al., 2022). PDT relies heavily on the presence of oxygen for its mechanism of action. Inadequate oxygen levels inside the tumor microenvironment, often known as tumor hypoxia, have been found to diminish the efficacy of PDT in *in vivo* systems. Tumor hypoxia is regulated by a variety of factors, including a high amount of the extracellular matrix, low pH levels, and immunosuppressive components resulting from disturbed metabolic processes and irregular tumor angiogenesis (Xiong et al., 2020; Mohammadi et al., 2021). Multiple studies have demonstrated that the presence of tumor hypoxia facilitates the proliferation of tumors (Li et al., 2020a; Anderson and Simon, 2020). Significant advancements have been made in the mitigation of tumor hypoxia through the administration of oxygen to tumors utilizing various oxygen carriers, including Hb oxygen carriers, non-Hb oxygen carriers, and hybrid proteins, among others (Luo et al., 2018; Wang H. et al., 2020). Nevertheless, the constrained loading efficiency and oxygen release of oxygen nanocarriers continue to pose a restricting constraint (Sun et al., 2020). In pursuit of this objective, there has been a heightened emphasis on the production of oxygen using nanomaterials, particularly those that have enzyme-like properties, referred to as “nanozymes.” The goal of these researchers is to address the issue of tumor hypoxia and make cancer therapies more accessible (Sutrisno et al., 2020). Various nanozymes, such as catalase (CAT), superoxide dismutase (SOD), oxidase (OXD), and peroxidase (POD) mimetic nanomaterials (Figure 1), have been employed to explore cancer therapies using two distinct therapeutic strategies: direct killing by enhancing ROS levels and indirect killing by depleting ROS (Zhang et al., 2020). The strategy of enhancing ROS levels has been found to enhance the effectiveness of therapy, particularly in the context of oxygen-dependent PDT, through the utilization of photosensitizer oxygenation enhancer (POD mimetics). This technique effectively addresses the challenge of tumor hypoxia. The POD mimetic nanozymes exhibit catalytic activity by facilitating the conversion of endogenous H_2_O_2_ into H_2_O and ROS within the tumor microenvironment. In spite of this, there is still a lack of exhaustive research on the application of POD mimicking nanozymes in the context of cancer treatments.

## 2 Enzymes and nanozymes

Enzymes serve as biocatalysts, playing a crucial role in facilitating the bulk of biological events that take place within living organisms (Garcia-Viloca et al., 2004). Nevertheless, the inherent limitations of natural enzymes, including their susceptibility to denaturation, expensive production, time-consuming preparation, and challenges in recycling, significantly restrict their feasibility for many practical uses (Lin et al., 2014). Since the 1950s, researchers have produced artificial enzymes as viable substitutes for natural enzymes in order to address the aforementioned constraints. These artificial enzymes offer enhanced stability and cost-effectiveness (Gao et al., 2007). Nanozymes have garnered significant attention in the last decade as a promising form of artificial enzymes. Nanozymes possess several notable benefits compared to both natural enzymes and traditional artificial enzymes (Table 1), including their ability to exhibit high and adjustable catalytic activities, cost-effectiveness, ease of large-scale manufacturing, and exceptional stability (Kotov, 2010). Nanozymes have garnered significant attention due to their numerous advantages. In the previous years, notable advancements have been made in the domain of nanozymes, coinciding with considerable progress in nanotechnology, biotechnology, and nanomaterial research (Korschelt et al., 2018; Wu et al., 2019).

**TABLE 1:** Nanozymes and natural enzymes comparison.

	Nanozymes	Natural enzyme
Advantages	- Significantly enhanced catalytic activity	- Significantly increased catalytic activity
	- Activity and types of catalysis that can be adjusted	- Great selectivity for the substrate
	- Mimetic activity of several enzymes	- A high level of biocompatibility
	- The great degree of stability	- Wide variety of biological catalysis
	- Use of recyclable materials	- Broad variety of possible uses
	- Simple for producing on a large scale	- Rational design through the manipulation of genes and proteins
	- Cost effective	
	- Storage for a very long time	
	- Resistance to hostile environments	
	- Multifunctionality is simple (big surface area for bioconjugation)	
	- The catalytic activity and various types of catalysts can be effectively regulated through the application of external stimuli, including but not limited to light, ultrasound, heat, magnetic fields, and other similar factors	
	- Distinct physicochemical characteristics, including fluorescence, electrical conductivity, and paramagnetic properties, that are exclusive to a particular substance	
Challenges	- There are a restricted range of nanozymes	- Excessively expensive
	- The substrate selectivity is restricted	- Insufficient stability
	- The unclear workings of the mechanism	-Difficult to store over the long term
	- Catalytic characteristics rely on structure, content, size, and shape	-It is difficult to make in large quantities
	- Insufficient levels of both standards and reference materials	-Separation and purification that takes up a lot of time
	- Nanotoxicity that could occur	-It is difficult to utilize in extreme conditions (such as high heat, extreme pH, high salinity, UV irradiation, etc.)

**FIGURE 2:**
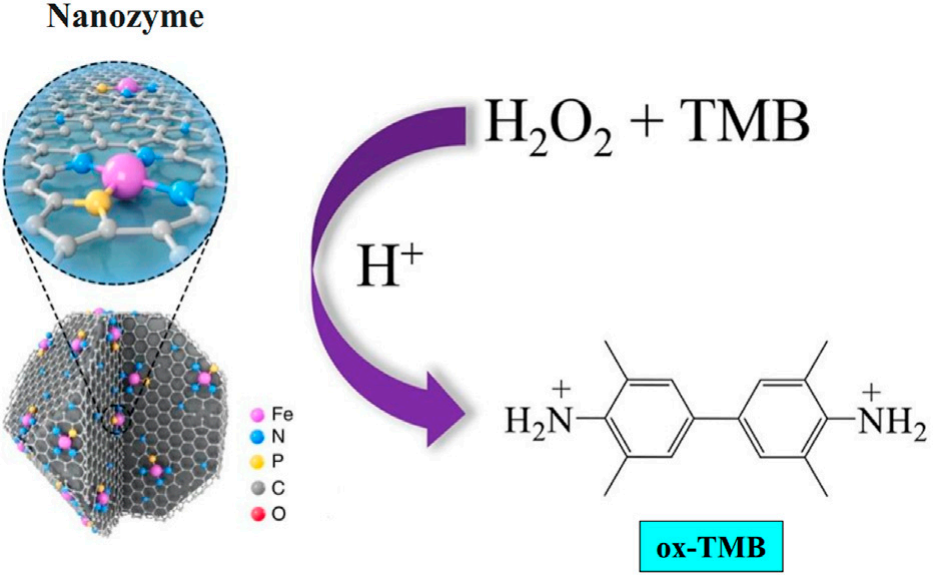
POD mimetic assay is depicted graphically.

Nanozymes are a type of nanomaterial with inherent enzyme-like characteristics. These properties are different from those observed in “synzymes” that involve real enzymes or the immobilization of catalytic ligands on nanomaterials. The name “nanozymes” was initially introduced by Pasquato and colleagues in 2004 to denote the transphosphorylation reactivity seen in gold (Au) NPs functionalized with triazacyclononane (Manea et al., 2004). The discovery of the inherent POD-mimic attribute of Fe_3_O_4_ NPs in 2007 led to the particular designation of nanozymes for nanomaterials possessing inherent enzyme-like properties (Gao et al., 2007). Research findings have indicated that Fe_3_O_4_ NPs possess the ability to facilitate the oxidation process of POD substrates, namely, O-phenylenediamine (OPD), diazo-aminobenzene (DAB), and 3,3,5,5-tetramethylbenzidine (TMB), in the presence of hydrogen peroxide (H_2_O_2_). This catalytic reaction leads to the generation of colorimetric responses, as depicted in Figure 2, thereby demonstrating the POD-mimic behavior of Fe_3_O_4_ NPs towards commonly encountered POD substrates. The catalytic reaction of the Fe_3_O_4_ nanozyme was found to follow a ping-pong mechanism, as determined by kinetics studies. According to the measured Michaelis-Menten kinetic characteristics, the Fe_3_O_4_ nanozyme had higher catalytic activity but lower substrate affinity than horseradish peroxidase (HRP) (Jiang et al., 2018).

This study will primarily examine the POD activities exhibited by nanoenzymes while also exploring the various parameters that influence the POD activity of these nanoenzymes. Subsequently, the utilization of nanoenzymes in the context of cancer diagnosis and treatment will be examined.

## 3 The POD mimic activity

As a group of enzymes called peroxidases work together with H_2_O_2_ to make reactive-free radicals. These radicals can react with a number of substrates, including TMB, OPD, and ABTS. To date, a range of nanostructures, including metal oxides, metals, metal sulfides, and carbon-based materials, have been observed to exhibit properties resembling those of POD enzymes (Yang et al., 2020). The initial demonstration of POD-like activity via a Ping-Pong mechanism was achieved by Fe_3_O_4_ NPs (Jiang et al., 2021). The oxidation process of TMB/H_2_O_2_ catalyzed by POD includes a two-step electron transfer mechanism. The initial step results in the formation of a TMB radical cation by a process of single-electron oxidation. When two intermediate radical cations come together, they make a blue charge-transfer complex that has absorption peaks at 370 and 652 nm. Additionally, in the next step of electron transfer, the cation radical goes through more oxidation, which creates a 450-nm-long TMB diamine derivative (TMBDI). The absorption spectra of the oxidation products of TMB typically exhibit three absorption bands. POD-imitated composite nanozymes exhibit comparable reaction kinetics as they facilitate the decomposition of H_2_O_2_ to generate ROS such as hydroxide ions and singlet oxygen. These ROS possess potential therapeutic applications. Several studies have identified a multitude of nanostructures with inherent POD-like activity. These NPs consist of a variety of substances, including fullerenes, polypyrroles, graphene oxide, carbon nanotubes, CdS, Co_3_O_4_, CeO_2_, Cu_2_O, FeS, CoFe_2_O_4_, FeTe, Fe_3_O_4_, FeSe, BiFeO_3_, MnFe_2_O_4_, and ZnFe_2_O_4_ (Attar et al., 2019).

## 4 Nanozyme catalytic activity can be influenced by many different factors

Although it is well accepted that NPs are typically considered to be biologically and chemically inactive, certain factors such as temperature, concentration, and pH can significantly impact the catalytic efficacy of nanozymes. In the subsequent part, we shall engage in a discussion concerning these parameters.

### 4.1 The effect of a medium’s pH on nanozymes activity

Wei and his colleague found that the rate at which ABTS was oxidized by H_2_O_2_ was faster when Fe_3_O_4_ NPs were present. This was true even when the solution was neutral or alkaline. Observations indicate that the presence of Fe^2+^ and Fe^3+^ ions in a solution can affect the breakdown of H_2_O_2_. Additionally, these ions have the potential to significantly increase the catalytic POD-mimic activity that nanozymes display. The leaching of Fe ions from Fe_3_O_4_ nanozymes in an acidic medium was indeed observed by Wei H. and colleagues. The data presented indicates that the produced nanozyme has significant activity in acidic pH conditions (Wei and Wang, 2008). Zhang and colleagues demonstrated the stability of Co_3_O_4_ NPs over a wide pH and temperature range. The stability of Co_3_O_4_ NPs as an inorganic nanozyme is considered to be superior to that of the natural POD (Mu et al., 2012). To validate this, Co_3_O_4_ NPs and HRP were subjected to incubation at various pH levels for a duration of 2 h. Subsequently, the activities of both entities were evaluated. HRP’s catalytic capability was significantly hindered following incubation at a pH below 4. Compared to the Co_3_O_4_ NPs, they exhibited sustained activity across a broad pH spectrum, ranging from 2 to 12. Co_3_O_4_ NPs are ideal for use in industrial applications due to their exceptional performance under challenging circumstances. Yang, Zhu, and colleagues investigated the effect of buffer solution pH level on the catalytic properties of Ag nanozyme. The catalytic studies were carried out in a buffer solution with different pH levels. The experimental results demonstrated that the pH values had a substantial impact on the relative activity of the Ag nanozyme. As the pH of the buffer solution changed to an acidic pH, catalytic activity increased (Liu et al., 2014).

### 4.2 The effect of the substrate concentration on the activity of nanozymes

Researchers have shown that there is a correlation between the catalytic activity of Ag nanozyme and the H_2_O_2_ concentration. The POD-mimic catalytic activity increases as the H_2_O_2_ concentration rises. At a concentration of approximately 2.2 mmol/L, which is 7.3 times more than that of TMB, the Ag nanozymes’ catalytic activity achieves its maximum value. However, the absorbance at 652 nm goes down as the H_2_O_2_ concentration goes above 2.2 mmol/L. This suggests that the catalytic activity goes down as the H_2_O_2_ concentration goes up. Indeed, nanozymes appear to exhibit a hump-shaped correlation between the concentration of the substrate and the activity of the reaction (Liu et al., 2014). The mechanism under discussion exhibits similarities to the one previously reported for HRP (Zhang and Toshima, 2013). It can be posited that the reactivity of the reaction initially increases as the concentration of H_2_O_2_ rises, as a greater amount of oxidant becomes involved in the reaction mechanism. The high concentration of H_2_O_2_ in the reaction system often leads to the idea that H_2_O_2_ molecules may stick to the catalyst surface, making it harder for other substances to stick to the nanozyme surface. As a result, this inhibition has a detrimental impact on the catalytic activity of the nanozymes. However, the catalytic activity based on the concentration of H_2_O_2_ differs from that of CuO NPs, Ag NPs, and Au NPs. In such instances, the observed reaction activity exhibits a hyperbolic relationship with respect to the concentration of H_2_O_2_ (Jv et al., 2010; Chen W. et al., 2012; Zhang and Toshima, 2013). One limitation of nanozymes is the elevated Michaelis-Menten constant (Km) associated with the reaction involving H_2_O_2_. This implies that a substantial concentration of H_2_O_2_ is required in order to effectively oxidize TMB. In one study, Huang et al. produced FeS_2_/SiO_2_ double mesoporous hollow spheres (DMHSs) and investigated its potential as an artificial POD. The experimental findings indicate that the vulcanization of Fe_3_O_4_ and the production of DMHS were successful approaches in improving the nanozyme’s affinity towards H_2_O_2_. The Km of FeS_2_/SiO_2_ dual-metal hybrid structures (with H_2_O_2_ as the substrate) exhibits a reduction of 18-fold compared to that of FeS_2_ NPs. The effectiveness of catalysis, as measured by the ratio of turnover number (Kcat) to Km, for FeS_2_/SiO_2_ DMHSs is approximately 16-fold greater than that of FeS_2_ NPs. The use of FeS_2_/SiO_2_ DMHSs to produce a nanozyme enables the sensitive and expeditious detection of H_2_O_2_ and glutathione within a time frame of 1 min at ambient temperature (Huang et al., 2020).

### 4.3 The impact of temperature on nanozymes activity

**FIGURE 3:**
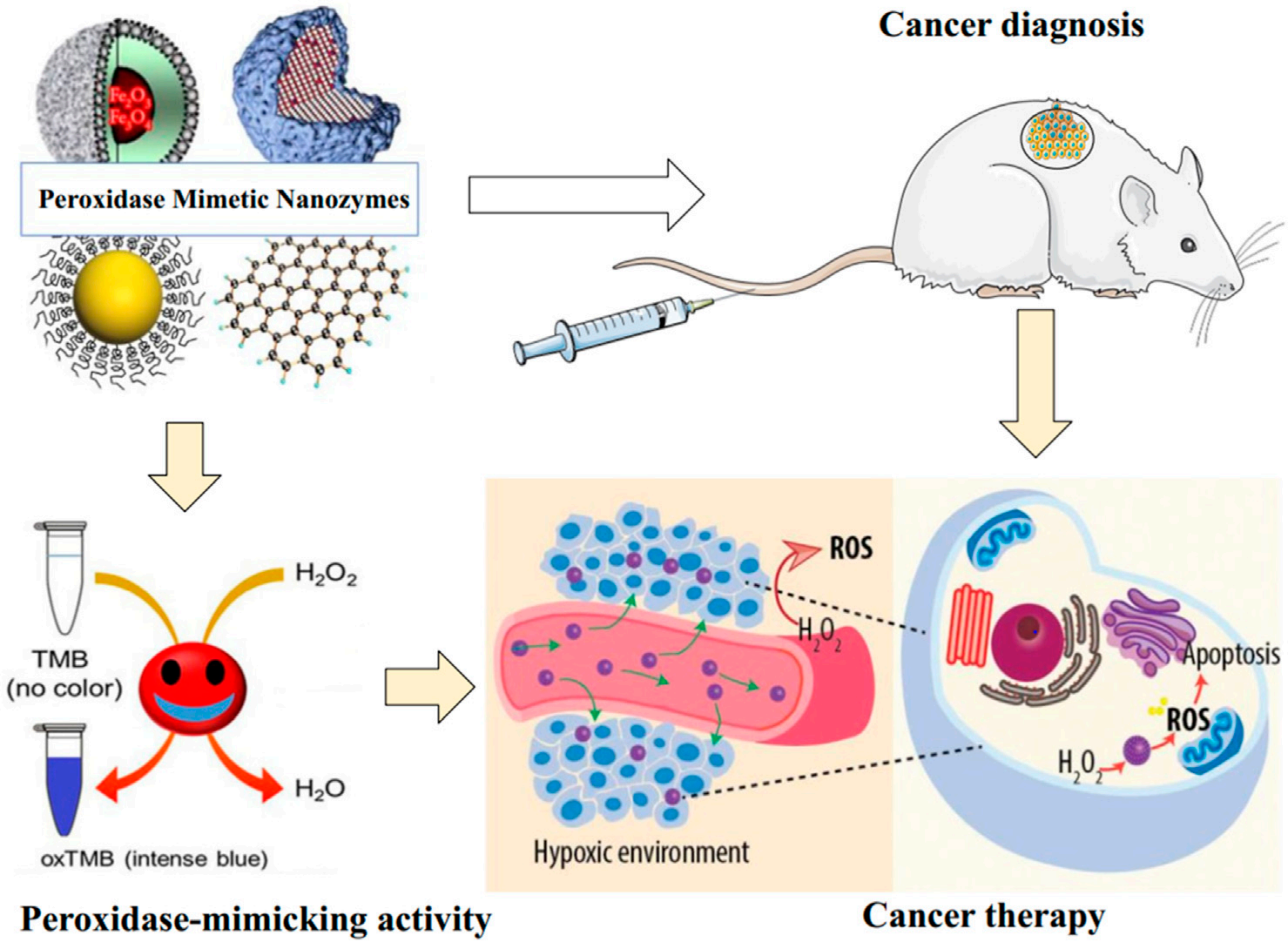
Schematic illustration of POD Mimic nanozymes activity and their applications in cancer diagnosis and therapy. The nanozymes combine POD-like activity and unique intrinsic properties of nanomaterials (e.g., paramagnetic properties). While some of their properties make them very suitable for imaging applications, the POD activity is used for the generation of toxic ROS resulting from H_2_O_2_ breakdown under hypoxic conditions in the TME.

A group of researchers investigated the POD-like activity of 300 nm Fe_3_O_4_ magnetic NPs at various temperatures. They then compared the obtained data with the POD activity observed in HRP under the same temperature conditions (Gao et al., 2007). The ideal temperature observed was roughly 40°C, a finding that closely aligns with the temperature ranges reported for HRP and other enzymes based on nanozymes (Zhang et al., 2010; Chen Z. et al., 2012). The investigation also looks into how temperature affects Ag nanozyme’s POD-like catalytic activity (Lu et al., 2015). The experimental results demonstrated that the optimal temperature for POD-like catalytic activity was found to be 25°C. The catalytic activity, which is influenced by temperature, exhibits similarities to that of natural enzymes or Fe_3_O_4_NPs, both of which demonstrate a specific temperature preference (Gao et al., 2007). Nanozymes, in comparison to natural enzymes, demonstrate enhanced thermal stability. Conversely, natural enzymes are frequently susceptible to adverse environmental conditions, leading to structural degradation and subsequent loss of enzymatic function (Wei and Wang, 2013). The application of these POD mimic nanozymes in the diagnosis and therapy of cancer, which was studied in the original articles, will be investigated further in the coming sections (Figure 3).

## 5 POD mimic nanozymes in cancer diagnosis

In the fight against cancer, early detection and therapy are important. An example of this is seen in patients with kidney cancer, where delayed detection significantly decreases survival rates. The survival rate for patients diagnosed with early-stage renal carcinoma is notably higher, reaching up to 99%, in contrast to the survival rate of patients diagnosed with advanced-stage renal cancer, which stands at 16% (Linehan et al., 2010). As a result, the primary benefit of early detection is an improvement in cancer survivorship, and creating early detection methods with high sensitivity and selectivity is crucial. On the other hand, precise tumor imaging is essential for several cancer treatments such as surgery, phototherapy, radiation therapy, and sonodynamic therapy (SDT). Nanozymes possess several advantageous characteristics, including cost-effectiveness, robust stability, exceptional catalytic activity, and versatile functionality. These attributes have facilitated their successful application in the identification of cancer-associated genes, molecules, and cells. Moreover, their inherent nanomaterial properties enable their utilization as probes for precise imaging purposes (Zhang et al., 2022) In a study conducted by Maji and colleagues, a novel nanostructured hybrid was synthesized with the purpose of serving as a mimetic enzyme for the *in vitro* diagnostic and therapeutic intervention of cancer cells. The mixed material, called GSF@AuNPs, was made by sticking AuNPs to very small pieces of reduced graphene oxide (rGO) that had mesoporous silica on top of them. Furthermore, the hybrid structure was augmented with folic acid, a ligand renowned for its strong attraction to cancer cells. The POD-like activity of the GSF/AuNPs hybrid was assessed by catalytically oxidizing a widely used POD substrate, TMB, in the presence of H_2_O_2_. This inquiry revealed previously unreported behavior. The hybrid probe was used to quickly, accurately, and selectively find cancer cells using colorimetry, taking advantage of its POD activity. Finally, the hybrid was employed as a mimetic enzyme for the purpose of cancer cell treatment, utilizing H_2_O_2_ and ascorbic acid (AA). In *in vitro* experiments, it was seen that HeLa cells were more likely to die when they were exposed to POD-mimic GSF@AuNPs along with either exogenous H_2_O_2_ or endogenous H_2_O_2_ produced by AA. This caused ROS (OH• radicals) to be produced. The experiment involved subjecting normal cells, namely, human embryonic kidney (HEK 293) cells, to treatment with a hybrid compound containing either H_2_O_2_ or AA. The findings of the study revealed that the hybrid compound exhibited a discerning cytotoxic effect on cancer cells (Maji et al., 2015). The levels of D-proline and D-alanine in the saliva of individuals in the early stages of gastric cancer exhibit a significant increase compared to those seen in the saliva of healthy individuals. Therefore, the careful surveillance of D-Ala and D-Pro levels carries substantial significance in the realm of gastric cancer diagnosis and treatment. In a study by Li Z et al., the pyrolysis of a glucose-filled ZIF-8 precursor (G@ZIF-8) produced carbon dots enclosed in N-doped carbon (CDs@NC). The CDs@NC NPs have POD-like nanozyme activity, which lets them be used for colorimetric detection of D-amino acids that are linked to early stomach cancer. The POD-mimic activity of CDs@NC can be easily changed by changing the amount of glucose precursors present, which is an interesting aspect. Additional investigation into the catalytic mechanism reveals that the superoxide radical (O^−2^ radical dot) potentially plays a crucial role as a ROS in POD-like catalytic processes. The promising outcomes obtained from saliva testing indicate that the colorimetric approach holds potential for rapid differentiation between individuals with early stomach cancer and those who are in good health (Li et al., 2022). Prussian blue nanozymes are a viable and affordable alternative to real POD in the ELISA (enzyme-linked immunosorbent test) due to the POD-like catalytic activity they exhibit. In a study, Khramtsov et al. propose a reliable method for functionalizing Prussian blue nanozymes. This process involves the trapping of nanozymes within albumin NPs. The process of adding monoclonal antibodies to NPs was made possible by cross-linking them with glutaraldehyde. The conjugates obtained were utilized as markers in an ELISA for the detection of prostate-specific antigen (PSA), a malignancy marker. The lower limit of detection was found to be less than 1 ng/mL, therefore allowing for the quantification of PSA within the range of quantities that are clinically significant (Khramtsov et al., 2022). Qu and colleagues have developed a novel colorimetric immunoassay for the detection of the cancer biomarker PSA. This assay utilizes the inherent POD activity of graphene oxide (GO). The GO has the ability to act as a catalyst in the interaction between H_2_O_2_ and hydroquinone, resulting in the formation of a solution with a brown color. In this research, the immunoassay used a secondary antibody (Ab2) that had graphene oxide added to it to make it stand out. Additionally, magnetic beads (MB) were chosen to immobilize the primary anti-PSA antibody (Ab1). When PSA is present, the immunocomplex formation process takes place. The antigen protein is placed between GO-Ab2 and MB-Ab1. By employing an external magnetic field, the immunocomplex was effectively isolated. Subsequently, varying quantities of GO-Ab2 were introduced into the solution, along with hydroquinone and H_2_O_2_, resulting in the manifestation of distinct hues. The visual perception of distinct hues can be utilized to immediately detect varying quantities of PSA. This straightforward immunoassay exhibits significant potential as a highly sensitive, specific, and point-of-care instrument for the clinical detection of cancer biomarkers (Qu et al., 2011). In one study, Song and colleagues present a novel composite material called folic acid-conjugated graphene-hemin (GFH). This mixture shows that it can quickly, accurately, and selectively find cancer cells through a colorimetric reaction made easier by GFH’s POD-like activity. The device has the capability to detect cancer cells at a minimum threshold of 1,000. In comparison to alternative techniques that depend on antibodies or natural enzymes, this composite demonstrates improved longevity and resilience against denaturation or degradation. Furthermore, the utilization of graphene in this system presents numerous benefits in comparison to alternative materials such as carbon nanotubes or mesoporous silica. The considerable dimensions of the GFH complex imply that it is observable using an optical microscope and can readily enter cells. As a result, the presence of cancer cells can be identified within cellular structures through the process of binding to surface receptors or other biomarkers. The strong π-π stacking interaction between graphene and hemin makes it possible for several hemin molecules to stick to both sides of a graphene monolayer. This arrangement ensures that each active site (hemin) is fully exposed to the substrates, leading to heightened activity. Graphene oxide (GO) also has a lot of functional groups, such as hydroxyl, epoxy, and carboxyl groups, which make it easier for biomolecules to stick to it in a flexible way. Consequently, our system exhibits considerable potential for various applications. The data obtained from this study would enhance the utilization of functionalized graphene derivatives in various applications, such as cell detection with high sensitivity and selectivity, analysis of DNA and proteins, and the development of novel imaging techniques (Song et al., 2011). Ultrasmall Au nanoclusters (AuNCs) are useful for imaging inside living things because they can collect in tumors and be flushed out of the body quickly by the kidneys. However, the natural catalytic activity of these substances, which could make detection more sensitive, has not yet been studied in the context of *in vivo* sensing. The writers, Loynachan et al., came up with multifunctional protease nanosensors that use the POD-like properties of AuNCs and the kidney’s precise filtration abilities at the nanoscale level. The nanosensors demonstrate the capability to react to disease microenvironments, leading to a urinary readout of the disease state through color changes. This readout can be achieved within a time period of less than 1 hour. The researchers kept an eye on the catalytic activity of AuNCs in urine samples from a colon cancer-bearing mouse model. The findings indicated that the mice afflicted with tumors demonstrated a 13-fold increase in the colorimetric signal in comparison to the animals that were in good health. The nanosensors were effectively removed by hepatic and renal excretion during a period of 4 weeks following injection, without any observed indications of harm. The authors anticipate that the use of this modular methodology will facilitate the expeditious identification of various diseases by leveraging their distinct enzymatic markers (Loynachan et al., 2019). The method Zhang and his colleagues used to make porous platinum (Pt) nanoparticles on graphene oxide (PtNPs/GO) was safe. Researchers have shown that the nanocomposite they made works well as a POD mimic, speeding up the reaction of a POD substrate when H_2_O is present. The researchers utilized PtNPs/GO as a signal transducer to create a colorimetric assay for the direct identification of cancer cells based on their POD-mimic activity. It is possible to tell the difference between 125 cancer cells (specifically MCF-7 cells) by looking at them directly. Folic acid is used as an identification element. The researchers have a vision that this particular nanomaterial possesses the ability to function as a versatile instrument in various applications within the fields of biotechnology and medicine (Zhang et al., 2014). In recent times, nanozymes have emerged as a highly promising tool for the application of cancer therapeutic approaches. Nevertheless, the ability to synthesize nanozymes with predictable sizes and implement targeting alterations remains a significant challenge in the field. Jiang et al. did a study on a HccFn(Co_3_O_4_) cobalt nanozyme that is based on ferritin and can selectively target and image hepatocellular carcinoma (HCC) tissues that are important for medicine. The biomimetic method is used to make the cobalt nanozyme, which is enclosed in the protein shell of the ferritin nanocage. This nanozyme has a high specificity for HCC. By genetically altering the ferritin surface to display the cell-specific peptide SP94, this targeting is possible. The nanozymes HccFn (Co_3_O_4_) have inherent POD-mimic activity, enabling them to catalyze substrates and induce a color reaction for the purpose of seeing HCC tumor tissues. The researchers conducted a study on 424 clinical HCC specimens. The researchers have verified that nanozymes composed of HccFn (Co_3_O_4_) have a high level of efficacy in distinguishing HCC tissues from healthy liver tissues. The sensitivity of this distinction was found to be 63.5%, while the specificity was determined to be 79.1%. These results are comparable to the diagnostic accuracy of the clinically employed HCC-specific marker, α-fetoprotein. The pathological study also suggests that the HccFn(Co_3_O_4_) nanozyme staining result could be used to help predict how well a patient with HCC will do in the future. The staining intensity of HCC patients has a positive correlation with tumor differentiation degree and tumor invasion, while demonstrating a negative correlation with overall survival. The results of this study show that ferritin is a very good building block for making nanozymes that can be different sizes and have specific changes made to them. Furthermore, the HccFn(Co_3_O_4_) nanozyme exhibits significant potential as a diagnostic reagent for prognostic evaluation of HCC (Jiang et al., 2019). MCF-7, as a breast cancer cell, demonstrates essential biological and developmental characteristics through the release of exosomes. Detecting the existence of breast cancer by analyzing exosome secretion in the bloodstream can serve as a reliable indicator. Nevertheless, the clinical application of exosome detection encounters obstacles in terms of accuracy and affordability. In a particular work, Long and colleagues developed an aptasensor utilizing nanozymes composed of iron oxide-copper ions (Fe_3_O_4_-Cu^2+^-NZs) and the CD63 aptamer. The CD63 aptamer was chosen because it can interact with exosomes. The Fe_3_O_4_-Cu^2+^-NZs were used because they have POD-like activity on TMB, which lets the nanozymes out. To look into how CD63 aptamer-Fe_3_O_4_-Cu^2+^-NZs interact with exosomes, exosomes had to be taken out of MCF-7 cells that had been grown. This research looked at how well CD63 aptamer-Fe_3_O_4_-Cu^2+^-NZs broke down TMB with and without exosomes. Analyzing UV-vis adsorption and visually evaluating color changes in both laboratory and real samples allowed for this. The results show that the POD-like activity of CD63 aptamer-Fe_3_O_4_-Cu^2+^-NZs was greatly reduced when exosomes were not present. This is likely because the aptamer coating was present. In ideal circumstances, the CD63 aptamer-Fe_3_O_4_-Cu^2+^-NZs construct exhibits the ability to identify exosomes within the concentration range of 1.4 × 10^4^–5.6 ×10^5^ particles/μL, with a minimum detectable concentration of 5.91 ×10^3^ particles/μL. Additionally, this approach demonstrated a favorable result in the identification of malignant cells in authentic specimens. In general, this colorimetric aptasensor demonstrates potential for the diagnosis of breast cancer cells through a straightforward and cost-effective methodology (Long et al., 2022).

Other studies in this topic are briefly summarized in Table 2.

**TABLE 2:** The application of POD mimic nanozymes in the field of cancer diagnosis.

POD mimic nanozymes	Nanozyme substrate	Method	Application	References
Antibody-conjugated	TMB	Colorimetric immunoassay (CI)	The detection of the HER2 protein	Kim et al. (2014)
Fe_3_O_4_/Pt NPs				
FeTIR NPs	TMB	PA	The utilization of *in vivo* imaging techniques for the visualization and characterization of tumors	Gong et al. (2020)
AuVCs NPs	HAuCl_4_ and H_2_O_2_	Lateral flow plasma sensing	The detection of glutathione (GSH)	Pang et al. (2020)
Pt@mSiO_2_ NPs	TMB	CI	The identification of the BRCA1 gene	Wang et al. (2014)
5mc-MIO NPs	TMB	CI and electrochemistry	The identification and measurement of global DNA methylation	Bhattacharjee et al. (2018)
PdCu@HRP NPs	TMB	CI	The detection of CEA glycoprotein	Huang et al. (2021)
GSF@AuNPs	TMB	CI	The identification and detection of cancer cells	Maji et al. (2015)
HccFn(Co_3_O_4_) NPs	DAB	Immunohistochemical assay	The identification of HCC cells	Jiang et al. (2019)

## 6 POD mimic nanozymes in cancer therapy

The utilization of nanozymes has been shown to demonstrate a wide range of functionalities within the realm of cancer treatment. Through the enhanced permeability and retention (EPR) effect, nanomaterials can carry drugs to tumors, making it easier for the drugs to target them passively. Nanozymes, similar to other categories of nanomaterials, exhibit the capacity to function as therapeutic agents in various forms of cancer treatment, encompassing, but not limited to, photosensitizers, sonosensitizers, and radiosensitizers. On the other hand, nanozymes exhibit distinctive characteristics like enzymes, which make them suitable for employment in catalytic therapy and starvation therapy. These applications facilitate the immediate elimination of tumor cells or the inhibition of tumor growth. Also, using nanozymes has shown that they can improve the effectiveness of cancer treatments in a number of ways, such as by increasing the delivery of oxygen (O_2_) and decreasing the levels of glutathione (GSH). In addition, the localization of therapeutic medications can be accomplished by leveraging the catalytic properties of nanozymes. This section elucidates the enzyme-mimetic attributes of nanozymes within the realm of cancer therapy (Zhang et al., 2022). While synergistic chemotherapy for tumors has been shown to have potential in treating cancer, it is still very hard to come up with a simple and effective way to build a multifunctional nanoplatform. The nanoformulation Au_2_Pt-PEG-Ce6 was developed by Wang et al. for the purpose of joint chemodynamic/phototherapy in the treatment of malignancies. In this formula, PEG is polyethylene glycol and Ce6 is chlorin e6. The current formulation talks about using Au_2_Pt nanozymes, which have activities similar to both catalase and POD. Additionally, the photosensitizer Ce6 is chemically bonded to the Au_2_Pt nanozymes to facilitate PDT. The nanoformulation described possesses the ability to generate O_2_ to alleviate tumor hypoxia and improve the efficiency of PDT. Additionally, it is capable of producing OH for chemodynamic therapy (CDT). Overall, Au_2_Pt-PEG-Ce6 demonstrates significant promise in the context of multimodal imaging-guided synergistic PTT, PDT, and CDT, showcasing notable tumor selectivity and improved therapeutic outcomes (Wang M. et al., 2020). The application of ultrasound-triggered SDT has garnered significant attention due to its potential for noninvasive treatment of massive interior tumors. In order to enhance the therapeutic outcomes of SDT, there is a need for the development of more efficient and durable sonosensitizers. Wang and his colleagues did a study in 2020 in which they made ultrafine titanium monoxide nanorods (TiO1+x NRs) that had much better sono-sensitization and Fenton-like catalytic activity. These nanorods were then employed to augment SDT. The TiO1+x NRs possess a highly refined rod-shaped morphology and have been subjected to surface modification using PEG. The PEG-TiO1+x NRs demonstrate nanozyme activity similar to HRP, allowing them to generate hydroxide ions through the reaction with endogenous H_2_O_2_ within the tumor. This capability enables the use of CDT. Unlike other sonosensitizers like TiO_2_ NPs, the PEG-TiO1+x NRs produced a lot more ROS when ultrasound was applied. This is because TiO1+x NR does not have a lot of oxygen in its structure. Its main job is to trap charges and stop the recombination of electron-hole pairs caused by ultrasound. Nanorods made of PEG-TiO1+x have shown promise as a sonosensitizer and CDT agent because they can effectively build up in tumors after being injected. This characteristic allows for highly efficient tumor ablation when subjected to ultrasonic treatment. Furthermore, the treated mice did not exhibit any notable long-term toxicity associated with PEG-TiO1+x NRs. This study showcases a novel titanium-based nanostructure that exhibits exceptional efficacy in the context of tumor SDT (Wang X. et al., 2020). Liang et al. conducted a study wherein they designed and synthesized a novel Pt-CuS Janus material consisting of hollow semiconductor CuS and noble metallic Pt. The copper sulfide (CuS) material exhibits a significant internal void that can be utilized for the encapsulation of sonosensitizer molecules, specifically tetra-(4-aminophenyl) porphyrin (TAPP), in order to facilitate the process of SDT. In addition, adding Pt NPs not only makes the photothermal performance better than CuS NPs because it increases the local electric field, but it also shows catalase- and POD-like properties. Because of these features, the Pt NPs can speed up the breakdown of endogenous H_2_O_2_ that is present in too high of a concentration in the body, creating O_2_. This O_2_ production can help overcome tumor hypoxia and enhance the generation of highly toxic ROS during SDT, leading to the effective apoptosis of cancer cells. It is important to note that shining an 808 nm laser on Pt-CuS makes heat, which improves the catalytic activity of Pt and raises the level of oxygen. This, in turn, promotes the effectiveness of SDT. It is noteworthy that the thermally responsive copolymer enveloping the Janus structure exhibits the capacity to function as an intelligent mechanism for adjusting the catalytic efficacy of Pt and governing the release of TAPP, thus exerting a substantial influence on the modulation of the therapeutic outcome. Together, synergistic catalysis, improved SDT effectiveness, and a very strong photothermal effect have made it possible to completely remove the tumor with a low chance of it coming back. Additionally, this approach has demonstrated a high level of therapeutic biosafety. In addition, the significant optical absorbance exhibited by the Pt-CuS Janus-produced material enables its application in photoacoustic (PA) imaging and near-infrared (NIR) thermal imaging. This study presents the development of a highly adaptable nanoplatform that can be utilized for a multifunctional theranostic approach. Additionally, the research expands the range of potential biological applications by employing a rational design methodology to optimize the structure of the nanoplatform (Liang et al., 2019). Gao et al. (2019) described the development of a highly effective biomimetic dual inorganic nanozyme-based nanoplatform. This nanoplatform was created to start a chain of catalytic events in response to the microenvironment of the tumor, which makes nanocatalytic tumor therapy possible. The platform used very small Au and iron oxide (Fe_3_O_4_) NPs that were loaded into dendritic mesoporous silica NPs at the same time. The gold nanoparticles can work like glucose oxidase, which means they can selectively turn β-D-glucose into gluconic acid and H_2_O_2_. Then, Fe_3_O_4_ NPs with POD-mimic activity catalyze the resulting H_2_O_2_. This catalytic process generates highly toxic OH, which induces cell death in tumor cells through a Fenton-based reaction. These biocompatible composite nanocatalysts have exhibited remarkable nanocatalytic-therapeutic efficacy, according to extensive *in vitro* and *in vivo* evaluations. These evaluations have further revealed a tumor suppression rate of 69.08%, which is highly desirable. Hence, this study establishes a pathway for the use of nanocatalytic tumor therapy through the deliberate creation of inorganic nanozymes possessing several enzymatic functions. This approach aims to achieve both a high level of therapeutic effectiveness and exceptional biosafety (Gao et al., 2019). Not many nanozymes stick to H_2_O_2_, and there is not much H_2_O_2_ in the tumor microenvironment (TME). This was the problem that Meng et al. wanted to solve in 2021. To tackle this challenge, they developed a pyrite POD nanozyme with an exceptionally high affinity for H_2_O_2_. It was amazing how much more catalytic activity this new nanozyme had compared to the classical Fe_3_O_4_ nanozyme and natural HRP—4144 times and 3,086 times, respectively. The researchers have made the discovery that the pyrite nanozyme possesses intrinsic glutathione oxidase-like activity, which enables the oxidation of reduced glutathione while concurrently producing H_2_O_2_. Therefore, the utilization of the dual-activity pyrite nanozyme presents a self-cascade mechanism for the production of a substantial amount of hydroxide ions and the depletion of reduced glutathione. This process leads to the induction of apoptosis and ferroptosis in tumor cells. As a result, it effectively eliminated tumor cells that were resistant to apoptosis and had a KRAS mutation by inducing ferroptosis. The pyrite nanozyme demonstrated good cytotoxicity specific to tumors and biodegradability, hence ensuring its biosafety. The findings of this study suggest that the pyrite nanozyme with high-performance characteristics demonstrates efficacy as a therapeutic agent, potentially contributing to the advancement of tumor catalytic therapy utilizing nanozymes (Meng et al., 2021). Furthermore, Li S. and colleagues reported the discovery of a new nanozyme (PtFe@Fe_3_O_4_) with dual enzyme-like capabilities, making it a promising candidate for highly effective tumor catalytic treatment. The PtFe@Fe_3_O_4_ composite has photothermal properties by nature, and it also has photo-enhanced POD-like and catalase-like activities in the acidic TME. Consequently, it demonstrates a remarkable ability to eradicate tumor cells and overcome tumor hypoxia. Significantly, the present study unveils a potential method for synergistic catalysis in PtFe@Fe_3_O_4_ through the utilization of photo-enhancement. The proponents have the belief that this endeavor will contribute to the progress of nanozymes in the field of tumor catalytic therapy (Li S. et al., 2019). Dong S. and colleagues have created a versatile nanozyme made of PEG/Ce-Bi@DMSN that possesses bacterial-like characteristics. We made the nanozyme by covering uniform Bi2S3 nanorods (NRs) with dendritic mesoporous silica (Bi_2_S_3_@DMSN) and then adding very small ceria nanozymes to the many mesopores of Bi_2_S_3_@DMSN. The nanozymes demonstrate two types of catalytic activities, resembling enzymes, namely, POD and catalase. These activities are observed under acidic conditions and have the ability to modulate the TME. Specifically, they can increase oxidative stress and alleviate hypoxia simultaneously. Furthermore, the nanozymes have a high capacity to efficiently metabolize the excessively produced GSH via a redox process. This study presents the utilization of photonic hyperthermia as a method to augment the dual enzyme-mimicking catalytic capabilities and reduce the excessive expression of GSH in malignant conditions, employing the technique of PTT. Getting this result is possible by using the PEG/Ce-Bi@DMSN nanozymes’ good light absorption in the second near-infrared (NIR-II) window. Treatment efficacy is greatly enhanced as a result of the subsequent enhancement of ROS-mediated mechanisms. Hence, this research work presents a demonstration of the feasibility of utilizing heat to enhance the multi-enzymatic capabilities of nanozymes in order to achieve tumor ablation (Dong et al., 2020). The utilization of immunomodulation-enhanced nanozyme-based tumor treatment strategies is widely regarded as a highly promising approach for the eradication of cancer cells. Xu and colleagues introduced a novel approach in an article, wherein they offered a strategy for tumor catalytic therapy using immunomodulation-enhanced nanozymes. This strategy aims to use the combined effects of nanozymes and TME regulation to obtain synergistic outcomes. The creation of TGF-inhibitor (TI)-loaded PEGylated iron manganese silicate NPs (IMSN), also known as IMSN-PEG-TI, is what initiates the therapeutic method. The findings of this work demonstrate that the IMSN nanozyme has inherent POD-like and catalase-like properties inside an acidic TME. These properties enable the nanozyme to effectively break down H_2_O_2_ into OH and O_2_, respectively. Moreover, it has been shown that both immunostimulatory nanomaterials (IMSN) and tumor immunotherapy (TI) have the ability to modulate the immunological microenvironment of tumors. This change causes macrophages to change from the M2 phenotype to the M1 phenotype, which makes it easier for H_2_O_2_ to grow again. The presence of H_2_O_2_ further enhances the catalytic activities of IMSN nanozymes. The efficacy of IMSN-PEG-TI in inhibiting tumor growth has been demonstrated by experiments conducted on multicellular tumor spheroids (MCTS) *in vitro* and *in vivo* models using CT26-tumor-bearing mice (Xu et al., 2020). In a work conducted by Zeng et al., a novel biodegradable boron oxynitride (BON) nanostructure was utilized to build a POD-like artificial enzyme. This artificial enzyme has shown high efficiency and multi-mode capabilities for breast cancer therapy. The BON nanozyme has catalytic activity in the generation of deadly OH, resulting in the induction of apoptosis in 4T1 cancer cells. This process leads to a substantial reduction in cell viability, with a decrease of 82% observed over a 48-h timeframe. The results of the *in vivo* experiment demonstrate that the BON nanozyme exhibits a significant level of efficacy in inhibiting breast tumor growth. Specifically, following a 14-day treatment period, the BON nanozyme was found to reduce tumor growth by 97% compared to the control group. In addition, it was demonstrated that the BON nanozyme exhibited a success rate that was tenfold higher compared to the inactive boron nitride (BN) nanospheres and 1.3 times superior efficacy when compared to the BN nanospheres that release boron. The findings of this study emphasize the significance of the BON nanozyme and its effective incorporation into the BN nanomedicine platform for the purpose of developing potent therapeutics for breast cancer (Zeng et al., 2021). Fans and other researchers have proposed a strategic approach for the coordination of nanozymes to specifically target tumor cells and carry out their activity in a selective manner to induce tumor destruction. Nitrogen-doped porous carbon nanospheres are used to make a nanozyme. These nanospheres have four enzyme-like functions (CAT, POD, OXD, and SOD) that are very important for controlling reactive oxygen species. After that, the researchers used ferritin to guide nitrogen-doped porous carbon nanospheres toward lysosomes. This increased the production of reactive oxygen species in a way that is only seen in tumors. This approach led to a notable regression of tumors in xenograft mouse models. Overall, the results of this study show that nitrogen-doped porous carbon nanospheres have important nanozymatic properties that make them good at controlling ROS inside cells. Additionally, the process of ferritinylation emerges as a highly encouraging approach for enhancing the tumor-targeting capabilities of nanozymes, hence facilitating their application in *in vivo* tumor catalytic therapy (Fan K. et al., 2018). The flexibility of NPs plays a crucial role in the assessment of cellular absorption and tumor accumulation, hence influencing the efficacy of cancer treatment. Wang et al. successfully made CuS-filled hollow nanocapsules that were embedded in human serum albumin (HSA). These were named CuS/HSA. In this study, the CuS/HSA hollow nanocapsules have a consistent size of 272.9 nm, a large hollow interior, POD-like properties, excellent photothermal conversion efficiency, and a large loading capacity for tetra-(4-aminophenyl) porphyrin (TAPP) at 27.3 wt%. The CuS nanoparticles had POD-mimic activity, which made them good at dealing with tumor hypoxia and improving the effectiveness of SDT effects and photothermal conversion in the context of PTT. It was shown *in vitro* that CuS/HSA-TAPP hollow nanocapsules can kill cancer cells by causing apoptosis under ultrasound (US) irradiation and destroying cancer cells under laser irradiation. This demonstrates the potential of these nanocapsules in SDT with PTT. Significantly, the cellular internalization of the CuS/HSA hollow nanocapsules was notably improved due to their flexibility, resulting in a longer mean residence duration of 131.3 h compared to their solid counterparts, which had a mean residence time of 21.0 h. According to the breast tumor model, the flexible CuS/HSA hollow nanocapsules showed a high rate of tumor growth of up to 27.1%. The efficacy of the CuS/HSA-TAPP hollow nanocapsules in eradicating breast cancers was established by *in vivo* tests, wherein the combined effect of SDT and PTT exhibited a synergistic outcome (Wang H. et al., 2022). Phthalocyanine is a macrocyclic ligand with conjugated properties that can operate as a metal ligand. It is anticipated to have a catalytic activity similar to POD enzymes, leading to the production of free radicals and the suppression of cancer cell growth. In one article, Wang et al. conducted a study where they synthesized phthalocyanine nanocrystals with various shapes using noncovalent self-assembly techniques within micro-emulsion droplets. The researchers utilized manganese phthalocyanine (MnPc) as the primary building block, which possesses a metal-N-C active core. The observed catalytic activity of these nano-assemblies is influenced by their form, since the co-mingling of the emulsifier and MnPc leads to a reduction in the compact arrangement of MnPc molecules, hence exposing a greater number of active sites. The assemblage possessed a nanostructure that was distributed in water, facilitating its accumulation at tumor locations by leveraging the EPR. The assembly exhibited enhanced catalytic activity exclusively in the acidic microenvironment, resembling tumors, owing to its very effective microenvironmental response. Importantly, this increased catalytic activity came with less harm to healthy tissues, making it a promising candidate for biological applications. The *in vivo* and *in vitro* catalytic treatment experiments have shown remarkable anti-tumor efficacy. This study investigated a novel approach for utilizing metal-organic macromolecules, specifically MnPc, as nanozymes in the context of catalytic tumor therapy (Wang J. et al., 2022). The biomedical profession has shown significant interest in graphene quantum dots (GQDs) that possess inherent enzyme-like properties. The primary emphasis of the application was directed towards the biosensing domain. However, graphene quantum dots’ (GQDs) insufficient catalytic efficiency restricted the investigation of anti-tumor properties. In this study, L. Hu et al. propose the utilization of graphene quantum dots/semiconducting polymer nanocomposites (GQD-SPNs) as an innovative approach to boost the activity of nanozymes for tumor therapy. This enhancement is achieved by utilizing the photothermal effect. The application of near-infrared irradiation induces a localized increase in temperature at the tumor site, hence enhancing the production of OH. This synergistic effect, in combination with the generated oxygen and heat, leads to improved efficacy in cancer therapy, specifically in Hela tumor-bearing mice. The current generation of graphene quantum dot-based supramolecular polymer nanocomposites (GQD-SPNs) has the potential to create significant opportunities for the advancement of nanozymes in cancer catalytic therapy (Hu et al., 2021). There is a study by Zhao et al. that shows how to make Fe_3_O_4_@Bi_2_S_3_ nanocatalysts (called F-BS NCs) that work like viruses. This involves the integration of Fe_3_O_4_ NPs (MNPs), known for their POD activity, with the narrow band gap semiconductor Bi2S3 (BS). The objective of this technique was to augment the enzymatic efficacy of the nanocatalysts through the utilization of restricted intratumoral peroxide and effective external photothermal stimulation. Based on the results of this study, the integrated F-BS NCs were found to effectively cause apoptosis in cancer cells with a mild photothermal treatment. Additionally, they demonstrate a sequential photothermal-stimulative catalysis of H_2_O_2_, resulting in the generation of extremely poisonous hydroxide ions species under an 808 nm laser. This synergistic approach successfully achieves a notable anticancer outcome (Zhao et al., 2020).

**FIGURE 4:**
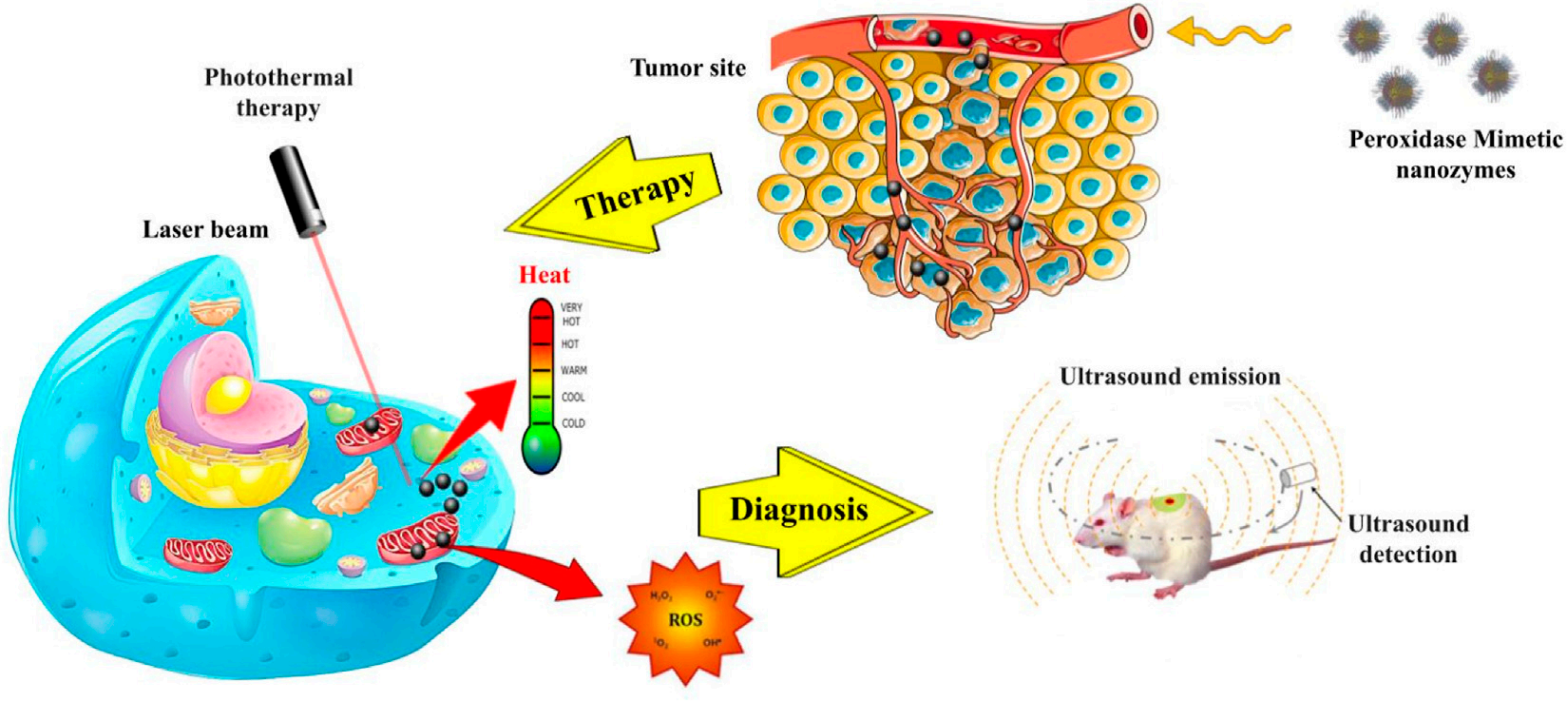
Schematic illustration of POD mimic nanozymes in oxidative and photothermal therapy and cancer diagnosis. These nanoparticles possess photothermal properties, which enable them to convert light into heat and thereby eliminate cancer cells. Additionally, they generate singlet oxygen atoms, which serve to augment the toxicity of the cells. In addition to their potential applications in cancer treatment, these nanozymes also exhibit promise in cancer diagnosis. They are capable of producing ultrasound waves by absorbing light due to their PA property.

Feng et al. developed a unique nanozyme, denoted as SFO (tin ferrite, SnFe_2_O_4_), that incorporates TME modulation for the purpose of enabling simultaneous PTT, PDT, and catalytic drug therapy (CDT). The SFO nanozyme, in its original state, exhibits both catalase-like and GSH POD-like activity. In the context of targeted molecular therapy, the initiation of H_2_O_2_ triggers the production of OH inside the TME. This process makes it easier to use CDT and lowers the amount of GSH inside cells, which makes tumors less able to fight free radicals. In the context of tumor hypoxia, the nanozyme exhibits the ability to catalyze the conversion of H_2_O_2_ into O_2_, hence improving the conditions for PDT (Figure 4). The SFO nanozyme also has a big phototherapeutic effect when it comes into contact with an 808 nm laser because it turns light into heat more efficiently (· = 42.3% more effectively) and makes very harmful free radicals. These researchers describe a nanozyme that works well as a tumor theranostic by permanently controlling the tumor microenvironment. It combines many types of treatment, such as computed tomography and magnetic resonance imaging. The implementation of this method has the potential to introduce a novel perspective in the development of alternative anticancer therapies based on tumor microenvironment engineering (Feng et al., 2021). In one study, Fang et al. developed a nanoscale Co-ferrocene metal-organic framework (Co-Fc NMOF) with notable Fenton activity. The researchers combined Co-Fc NMOF with glucose oxidase (GOx) to make a cascade enzymatic/Fenton catalytic platform (Co-Fc@GOx). This made it even more likely that it could be used to treat tumors. The Co-Fc NMOF in this system shows that it can do many things and is very good at carrying GOx molecules, which lets the reaction conditions be changed. Additionally, it exhibits an exceptional Fenton effect, leading to the production of highly poisonous OH. GOx is delivered to the TME through Co-Fc NMOF, and it speeds up the process of turning glucose into H_2_O_2_ and gluconic acid. It makes the inside of cells more acidic and raises the concentration of H_2_O_2_ on the site, which then helps the Fenton reaction of Co-Fc NMOF and increases the production of ROS. The amazing anticancer capabilities of the cascade enzymatic/Fenton catalytic process triggered by Co-Fc@GOx nanozyme have been demonstrated in both *in vitro* and *in vivo* experiments (Fang et al., 2020). Other studies in this topic are briefly summarized in Table 3.

**TABLE 3:** The utilization of POD mimic nanozymes in the field of cancer therapy.

POD mimic nanozymes formulation	Nanozyme substrate	Activity	Application	References
PCF-a NANOPARTICLE	H_2_O_2_, GSH	POD, GPx	The depletion of glutathione (GSH) to increase ultrasound and near-infrared (NIR)-promoted cancer drug therapy (CDT)	Jana et al. (2021)
PtCu3 nanocages	H_2_O_2_, GSH	POD, GPx	The reduction of glutathione (GSH) in order to increase cellular DNA damage response (CDT)	Zhong et al. (2020)
TMPAs	TMB	POD	Nanozyme-amplifed NIR-II PTT	Ma et al. (2021)
MnPcNPs	H_2_O_2_	POD	Catalytic treatment	Wang et al. (2022b)
Pd—C SAzymes	H_2_O_2_	POD	Catalytic H_2_O_2-_supply treatment	Zhu et al. (2022)
DMSN-Au-Fe_3_O_4_	Glucose, H_2_O_2_	POD, GOx	OXD-POD cascade catalytic therapy	Zhu et al. (2022)
pyrite NPs	GSH, H_2_O_2_	GSHOx, POD	OXD-POD cascade catalytic therapy	Meng et al. (2021)
IAA-loaded PNCNzyme		IAA	Chemotherapy with nanozymes	Liang et al. (2020)
Au2Pt-PEG-Ce6		CAT, POD	Phototherapy and Chemodynamic therapy	Wang et al. (2020b)
SFO nanozyme		CAT, POD	Synergistic phototherapy	Feng et al. (2021)
Co_9_S_8_ NDs		POD	PDT/PTT	Lin et al. (2018)
FeN200@GOx@M		POD	a combined treatment strategy (UTMD and enzyme)	Xiang et al. (2021)
GQD-SPNs		POD	Cancer catalytic therapy was improved by PTT	Hu et al. (2021)
PDAC NPs		POD	CHT/CDT/PTT combination therapy	Xu et al. (2019)
FeΙΙΙ-doped C_3_N_4_ nanosheets			PDT guided by MRI	Ma et al. (2016)
AgPd@BSA/DOX		POD, CAT	chemotherapy/hyperthermia/ROS	Li et al. (2020b)
PCN-224-Pt		POD	PDT	Zhang et al. (2018)
PB-Ft NPs		POD	Chemo-PTT through ROS generation	Li et al. (2019b)
ABTS@PAH-CNts		POD	PTT	Chen et al. (2021)
Au@HCNs		POD and OXD	PTT catalyzed by enzyme	Fan et al. (2018b)
Au-Ag@HA NP		POD	Combination therapy with radiation, nanozymes, and Ag^+^	Chong et al. (2020)
ZIF-8 NPs coated with Ce6 and Cyt c		CAT, POD	Protein treatment and PDT	Mahalunkar et al. (2019)

## 7 Conclusion

Since the discovery of ferromagnetic NPs as effective substitutes for natural enzymes in 2007, nanozymes have garnered significant attention and found numerous applications, particularly in the field of oncology. Despite the significant advancements made in various domains by nanozymes, there remain certain noteworthy challenges that demand attention. At the moment, most of the research in the field of nanozymes is focused on studying oxidoreductase and hydrolase activities. Other enzyme activities, like transferase and lyase, are still not well understood. Hence, it is imperative to investigate novel nanozyme materials and thoroughly examine their catalytic characteristics. Furthermore, the catalytic mechanism of nanozymes exhibits a wide range of diversity and is subject to regulation by multiple circumstances. Furthermore, it is worth noting that several nanozymes have the potential to exhibit a synergistic impact throughout the process of combating tumors. Hence, it is imperative to ascertain the comprehensiveness of various nanozyme catalytic systems. The present catalytic efficiency of nanozymes poses challenges in attaining the *in vivo* performance level of natural enzymes. Furthermore, the activities of nanozymes remain constrained by the intricate TME. Furthermore, the issue of inadequate substrate selectivity in nanozymes remains unresolved. Nanozymes can be used to change certain molecules, which could help solve the problem we talked about earlier because they could improve substrate specificity and make targeting tumors more accurate and sensitive. Furthermore, investigations into the application of nanozymes in tumor theranostics are currently in the nascent phase. These materials’ inherent toxicity and clearance rate are limiting their wide range of applications. Furthermore, it is important to note that different nanozymes possess distinct advantages and limitations. Hence, the imperative objective would be to fabricate a nanosystem that exhibits commendable biocompatibility, superior targeting efficacy, and multifaceted functionalities. The field of nanoscale science and technology has witnessed significant advancements, leading to the emergence of nanozymes. These nanozymes exhibit remarkable versatility, operability, and applicability, thereby opening up new avenues for principles and technologies in disease diagnosis and treatment. Additionally, they offer promising prospects for the development of efficient and precise nanodrug applications in the biomedical domain.
